# Association of Night Shift Work and Related Risk Factors with Overweight and Obesity across Europe: The SHIFT2HEALTH Online Survey

**DOI:** 10.1159/ofa/adzag009

**Published:** 2026-09-12

**Authors:** Sandra Haider, Monique H. Vingerhoeds, Maria Wakolbinger, Kyriaki Papantoniou, Isabel Santonja, Katrin Scionti, Vanessa Schoissengeier, Karl-Heinz Wagner, Hannah Jilani, Urte Klink, Katarzyna W. Binder-Olibrowska, Jarosław Rakoczy, Magdalena Agnieszka Wrzesińska, Coen Dros, Heidi Lammers-van der Holst, Meeke Ummels, Marlies Wallner, Anna Lena Aufschnaiter, Christina Höfler, Bianca Fuchs-Neuhold, Daniela Grach, Catalina Cuparencu, Logan Stuck, Hendriek C. Boshuizen, Imke Mattulat, Edith Feskens, Eva Winzer

**Affiliations:** a Department of Social and Preventive Medicine, Centre for Public Health, Medical University of Vienna, Vienna, Austria; b Wageningen Food and Biobased Research, Wageningen University and Research, Wageningen, The Netherlands; c Department of Epidemiology, Centre for Public Health, Medical University of Vienna, Vienna, Austria; d Barcelona Institute for Global Health (ISGlobal), Barcelona, Spain; e Department of Nutritional Sciences, University of Vienna, Vienna, Austria; f Vienna Doctoral School of Pharmaceutical, Nutritional and Sport Sciences (PhaNuSpo), Vienna, Austria; g Institute for Public Health and Nursing Research-IPP, University of Bremen, Bremen, Germany; h Department of Psychosocial Rehabilitation, Medical University of Lodz, Lodz, Poland; i Department of Public Health, Erasmus MC, Rotterdam, The Netherlands; j Institute of Dietetics and Nutrition, FH JOANNEUM University of Applied Sciences, Graz, Austria; k Department of Nutrition, Exercise and Sports, University of Copenhagen, Copenhagen, Denmark; l Biometris, Plant Science Group, Wageningen University and Research, Wageningen, The Netherlands; m ttz Bremerhaven, CF Sensory and Consumer Research, Bremerhaven, Germany; n Division of Human Nutrition and Health, Wageningen University and Research, Wageningen, The Netherlands

**Keywords:** Obesity, Overweight, Night shift, Lifestyle parameters

## Abstract

**Introduction:**

Night shift work is associated with higher risk of overweight/obesity (OW/OB). This study examines the prevalence of OW/OB among current and former night shift workers, compared to day workers, across Europe, and assesses the influence of night shift metrics and lifestyle/psychosocial factors on this relationship.

**Methods:**

Data on participants’ self-reported BMI, night shift metrics, sociodemographic, and lifestyle/psychosocial factors were collected via an online survey in Austria, Denmark, Germany, Greece, Italy, the Netherlands, Poland, and Spain (May 2024–January 2025). Logistic regression models analysed the associations between night shift work metrics, lifestyle/psychosocial factors, and OW/OB, accounting for additional adjustments.

**Results:**

Among 6,148 Europeans (mean age: 40.6 [10.6] years; 50% female), OW/OB prevalence was highest in Germany and lowest in Denmark (61% vs. 41%; *p* < 0.001). Current and former night shift workers exhibited higher prevalence of obesity (OR 1.62, 95% CI: 1.33–1.99; OR 1.51 95% CI: 1.19–1.93) than day workers. Longer, more frequent, and rotating night shift exposure correlated with higher odds of OW/OB. Among current night shift workers, factors such as fast eating rate, physical inactivity, and moderate/high alcohol consumption influenced OW/OB prevalence, with unfavourable dietary patterns linked to OB. Adjustments for lifestyle/psychosocial factors had minimal impact on these outcomes.

**Conclusion:**

Current and former night shift workers exhibited a higher prevalence of OW/OB than day workers. Lifestyle factors, especially eating rate and physical activity, partly explain this elevated risk. Therefore, prevention strategies could benefit from combining organizational, environmental, and workplace interventions, particularly since an elevated BMI may persist even after night shift work has ceased.

## Plain Language Summary

This study looks at how often overweight/obesity affects people who work night shifts or day shifts in several European countries. It also looks at how work schedules, lifestyle, and mental health factors influence this. Information was collected from 6,148 people aged 40.6 years on average via an online survey conducted in Austria, Denmark, Germany, Greece, Italy, the Netherlands, Poland, and Spain between May 2024 and January 2025. The results showed that the participants from Germany had the highest overweight/obesity rate (61%), while those from Denmark had the lowest (41%). People who currently or previously worked night shifts were more likely to have overweight/obesity compared to people who worked during the day. This was particularly the case for people who worked night shifts more often, for longer, or in a rotating pattern. The lifestyle factors that were linked to the higher risk of developing overweight/obesity included fast eating, physical inactivity, and moderate-to-high alcohol consumption. It is notable that lifestyle and mental health factors explained only a small part of the increased risk that was seen. The results show that people who work nights or used to work night shifts are more likely to have a higher BMI. This means that changes to how they work and the environment they work in need to be made to reduce these risks. This is because a higher BMI can last even after people stop working nights.

## Introduction

Over the past 30 years, the prevalence of overweight/obesity (OW/OB) has risen sharply worldwide; now over half of the adults in Europe live with OW/OB [1]. Among many factors, occupational exposures and particularly night shift work have been identified as important contributor to the increasing prevalence of obesity [2, 3]. In 2024, 18% of employees reported working nights, according to data from Eurostat, with rates ranging from 7% in France to 41% in Serbia [4]. Meta-analyses show that night shift work was associated with a 17–25% higher risk of OW/OB, compared to day work [5]. However, effect sizes vary by population (e.g., working sector) and country [3, 6]. For example, among nurses, no significant overall association was observed in the pooled estimate of 11 studies, although subgroup analyses indicated regional differences with a 36% increased risk in North America compared to a 1% increase in Europe and Australia [6]. More detailed analyses suggest that shift work metrics such as the intensity and type of shift work exposure matter; for example, Sun M et al. [3] found an exposure-response relationship between night shift duration (years of exposure) and intensity (number of night shifts per month) and OW/OB risk, although quantification was not possible due to the limited number of studies and the heterogeneity of intensity measures. Furthermore, in the CONSTANCES cohort, obesity risk was elevated among permanent and especially rotating night shift workers, compared to day workers, while risks were elevated but lower among former night shift workers [7].

Higher OW/OB rates among night shift workers may be due to circadian disruption and sleep impairments and altered diet and lifestyle which impairs insulin sensitivity, energy expenditure, immune and hormonal function [8, 9]. Also, altered lifestyle factors, such as an unfavourable diet and physical inactivity, may play a role [10, 11]. Nurses on rotating night shifts were shown to consume fewer fruits and vegetables and more savoury snacks, sugar-sweetened beverages, and energy-dense foods high in fat and sugar [12–15]. Leisure-time physical activity was also studied, but evidence is inconclusive; a meta-analysis found no significant differences between former and current night shift workers compared to day workers [16], whereas evidence from the Finnish public sector suggests that night shift workers are less active than day workers [17]. Additionally, sleep quality is generally poorer among night shift workers with fixed and rotating shifts compared to day workers [18], and night shift work is also associated with worse mental health outcomes including higher levels of psychological distress [19].

There is a lack of Europe-wide, comprehensive studies using consistent methodology to examine the association between night shift exposure and OW/OB, which limits cross-country comparisons. Additionally, research on the associations between former night shift work as well as detailed shift work metrics and BMI is sparse. Therefore, this study aimed to compare current and former night shift workers with day workers [1], examine the prevalence of OW/OB (based only on self-reported BMI) in shift workers and day workers in eight European countries [2], assess the associations between work schedule metrics (years of night shift exposure, frequency of night shifts, rotating or fixed night shifts, and years since last night shift) and lifestyle parameters (eating rate, dietary patterns, physical activity, insomnia problems, alcohol consumption, and smoking status) and psychosocial status (perceived stress level and mental well-being), in relation to OW/OB, and [3] examine the extent to which lifestyle or psychosocial factors modulate the association between shift work exposure and OW/OB.

## Methods

### Study Design

As part of the European Union-funded SHIFT2HEALTH project (https://shift2health.eu/), an online survey was performed across eight European countries (Austria, Denmark, Germany, Greece, Italy, the Netherlands, Poland, and Spain) between May 2024 and January 2025 to obtain a broad overview of lifestyle factors and current working conditions. Study data were collected and managed using Research Electronic Data Capture (REDCap) electronic data capture tools hosted at the University of Vienna [20, 21]. REDCap is a secure, web-based software platform designed to support data capture for research studies, providing (1) an intuitive interface for validated data capture; (2) audit trails for tracking data manipulation and export procedures; (3) automated export procedures for seamless data downloads to common statistical packages; and (4) procedures for data integration and interoperability with external sources. The meta-dataset is available from the Zenodo repository (https://doi.org/10.5281/zenodo.17423134).

### Recruitment and Inclusion Criteria

Recruitment took place through various channels, including online platforms (LinkedIn, Facebook, Instagram), newsletters from the European Institute of Innovation & Technology (EIT) Health, company partnerships, personal networks, and with the help of a recruitment agency. The study applied a convenience and voluntary response sampling strategy, further supplemented by snowball recruitment through participant referrals and social media sharing. The aim was to recruit a total of 4,800 participants (at least 400 night shift workers and 200 day or former night shift workers per country). In order to be eligible to participate, individuals had to be aged ≥21 years, be currently employed or self-employed, and work ≥28 h per week.

### Questionnaires

The following questionnaires were provided in eight languages. If no validated version was available, forward and backward translation and pretesting were carried out [22].

#### Self-Reported Data on BMI

BMI was calculated from self-reported height and weight and values were classified into (1) normal weight (≤24.9 kg/m^2^); (2) overweight (25.0–29.9 kg/m^2^); and (3) obesity (≥30.0 kg/m^2^) [23].

#### Sociodemographic Data

Questions on age, gender, country of residence, income satisfaction, and marital status were based on the European Social Survey (ESS) [24] and the EU Statistics on Income and Living Conditions (SILC) [25]. Education level was assessed using the International Standard Classification of Education (ISCED) 2011 levels [26].

#### Night Shift Exposure

Participants’ occupations were assessed using predefined categories derived from the Austrian Public Employment Service [27]. Based on a consensus paper [28], night shift status was assessed using the question, “*have you ever worked night shifts (schedule including ≥3 h of work between midnight and 5.00 a.m.)?*”. The answer options were (1) no; (2) yes, in the past; and (3) yes, currently. All participants were asked to report their current working schedule in detail, by providing start and end times of their four most frequent shifts. Current night shift workers were also asked about their years of night shift exposure, frequency of night shifts/month, and the direction of shift rotations, with years of exposure and monthly frequency categorized into tertiles. Direction of shift rotations was categorized as either fixed (always the same working hours) or rotating (varying working hours). Former night shift workers also reported the years of their night shift exposure, the direction of shift rotations, and the years since their last night shift, with years of exposure and time since last night shift categorized into tertiles. Work sectors were categorized into 15 different sectors and subsequently dichotomized into “health, social, and beauty care” or “other” sectors (including trade, logistics and transport, as well as office, marketing, finance, law, and security).

#### Health Conditions

Information on self-reported health conditions over the last 12 months was collected. A total of 21 health conditions were queried, and those with a prevalence of more than 10% or significant group differences are shown.

Lifestyle and psychosocial parameters including eating rate, dietary patterns, physical activity, insomnia problems, alcohol consumption, and smoking status as well as perceived stress and mental well-being were assessed across several questions: eating rate was assessed by the question, “*how would you describe the speed at which you eat compared to others?*” with the answer options (1) very slow; (2) slow; (3) average; (4) fast; and (5) very fast [29]. We dichotomized eating rate as slow/average [1–3] and fast [4, 5].

Dietary patterns were assessed using six items derived from the validated 13-item rapid Prime Diet Quality Score screener (rPDQS) [30]. Participants reported their usual consumption frequency of six food groups over the past month: (1) sugar-sweetened beverages (e.g., soda, soft drinks, sports drinks); (2) refined grains (e.g., white bread, rice, pasta); (3) whole grains (e.g., bread, breakfast cereals, brown rice); (4) legumes and pulses (e.g., beans, chickpeas, tofu); (5) vegetables (e.g., carrots, spinach, broccoli, NOT potatoes); (6) whole fruits (e.g., apples, oranges, bananas). Response options were (1) ≤1×/week; (2) 1×/week; (3) 2–4×/week; (4) nearly daily or daily; and (5) ≥2×/day. A composite dietary pattern score (0–12) was calculated using a “traffic light” coding system (green/most favourable = 2 points, yellow/moderate = 1, and red/least favourable = 0) reflecting intake frequency of each food group. For example, vegetable intake was coded as red for response options 1–3, yellow for option 4, and green for option 5 [30]. Total scores were dichotomized based on the median split into “less favourable” (0–5) and “more favourable” [6–12] dietary patterns.

Physical activity was assessed using the Godin Leisure-Time Exercise Questionnaire [31], asking the question, “*during a typical 7-day period, how many times on the average do you do the following kinds of exercise for more than 15 min during your free time?*”. Respondents were asked to report the time they spent doing vigorous, moderate, or light exercise, and were classified as insufficiently active (<14 points) and active/moderately active (≥14 points).

Insomnia problems were assessed using the first three items of the Insomnia Severity Index (ISI) [32], which cover sleep onset, sleep maintenance, and early morning awakening. Items were rated on a five-point Likert scale, ranging from 0 (none) to 4 (very severe insomnia problems) and *s*ummed up to a total score. We dichotomized the scores as no to moderate (<4 points) and severe insomnia problems (≥4 points).

Alcohol consumption was assessed using questions (with minimal modifications) from the EHIS [33], asking, “*how many drinks/glasses do you consume in an average week when you drink alcohol?*”. Response options were 0, 1, 2, 3, 4–5, 6–9, 10–15, and 16 or more drinks/glasses per week. For analyses, alcohol consumption was dichotomized as no/low consumption (0–2 drinks/glasses per week) versus moderate/high consumption (≥3 glasses per week).

Smoking history was assessed using the question “*do you currently smoke any tobacco products? (cigarettes, cigars, or pipes)*” and *“have you ever smoked tobacco products?”* [33]. Participants who reported current smoking were classified as current smokers, while those who reported no current smoking but had smoked previously were classified as former smokers. For analyses, participants were categorized as either never smokers or smokers (current and former). Perceived stress was assessed using the Perceived Stress Scale 4 (PSS-4), including four items on a five-point Likert scale with a sum score ranging from 0 to 16 [34]. A score of ≥6 indicated high stress [35].

Mental well-being was assessed using the WHO-Five Well-Being Scale (WHO-5), a self-reported instrument consisting of five statements referring to how an individual has been feeling over the past 2 weeks. A score of ≤50 points indicated poor mental well-being [36].

### Statistical Analysis

Continuous variables are presented as means and standard deviations (SDs), and categorical variables as numbers and percentages (%). Multivariable logistic regression analyses were conducted to examine the association between night shift work exposure (current night shift workers, former night shift workers, and day workers) and OW/OB (reference category: normal weight). In addition, logistic regression analyses were performed, stratified by night shift work status, to assess the association between work schedule matrix and OW/OB. All regression models were adjusted for age, gender, sociodemographic variables (country, education level, income satisfaction, and marital status), and occupational factors (hours worked per week and current job duration), with covariates selected based on existing literature. Further comparable multivariable logistic regression models were calculated to examine the associations between lifestyle/psychosocial parameters and OW/OB, stratified by night shift work status, and adjusted as described above. To examine the potential contribution of lifestyle/psychosocial factors to the association between night shift work and OW/OB, lifestyle/psychosocial factors (including eating rate, dietary patterns, physical activity, insomnia problems, alcohol consumption, smoking status, perceived stress level, and mental well-being) were subsequently added to the model determining the association between shift work and OW/OB. Odds ratios (ORs) and 95% confidence intervals (CIs) were reported. The level of statistical significance was set at *p* < 0.05. Statistics were computed using IBM^®^ SPSS^®^ Statistics Version 30 (IBM Corp., Armonk, NY, USA).

#### Sensitivity Analyses

To test the robustness of night shift work classification, we calculated agreement between the question, “*have you ever worked night shifts?*” and a classification based on detailed working schedules using Cohen’s kappa statistic within the three groups.

## Results

### Characteristics of the Study Population

A total of 21,316 individuals initiated the survey, of whom 20,835 provided informed consent (online suppl. Fig. 1; for all online suppl. material, see https://doi.org/10.1159/ofa/adzag009). A total of 8,102 participants fulfilled the inclusion criteria and provided data on sociodemographic characteristics and work schedule. After further excluding individuals with missing data on BMI and lifestyle factors, 6,148 participants remained. The final data set included 49.7% women, with a mean age of 40.6 (SD: 10.6) years; 43.9% had a university degree and 21.9% reported difficulty coping on their current income (Table 1). Forty-one percent worked in the “health, social, and beauty care sector” with the rest in trade, logistics, office, finance, and other sectors. However, the distribution differed between groups, with current night shift workers more frequently employed in the “health, social, and beauty care sector” (50.8%) compared to former night shift workers (29.2%) and day workers (20.6%). The most common reported health conditions were migraine (18.8%), high cholesterol or triglycerides (17.8%), hypertension (17.2%), and atopic episodes (11%).

**Table 1.** Characteristics of the study sample, stratified by night shift work exposure

​	Current night shift workers *n = *3,697 (60.1%)	Former night shift workers *n =* 1,274 (20.7%)	Day workers *n =* 1,177 (19.2%)	Total *n = *6,148 (100%)
Age, years	40.5 (10.7)	40.9 (10.3)	40.4 (10.7)	40.6 (10.6)
Gender				
Female	1,772 (47.9%)	603 (47.3%)	680 (57.8%)	3,055 (49.7%)
Male	1,911 (51.7%)	668 (52.4%)	493 (41.9%)	3,072 (50%)
Non-binary, others	12 (0.4%)	2 (0.2%)	4 (0.3%)	18 (0.3%)
Prefer not to answer	2 (0.1%)	1 (0.1%)	0 (0%)	3 (0%)
Education				
Basic education not completed	14 (0.4%)	11 (0.9%)	5 (0.4%)	30 (0.5%)
Basic education	237 (6.4%)	92 (7.2%)	58 (4.9%)	387 (6.3%)
High school and vocational training	903 (24.4%)	310 (24.3%)	297 (25.2%)	1,510 (24.6%)
Advanced training and college	942 (25.5%)	330 (25.9%)	230 (19.5%)	1,502 (24.4%)
University degrees and beyond	1,591 (43%)	527 (41.4%)	583 (49.5%)	2,701 (43.9%)
Unknown/prefer not to say	10 (0.3%)	4 (0.3%)	4 (0.3%)	18 (0.3%)
Income satisfaction				
Living comfortably	1,207 (32.6%)	332 (26.1%)	318 (27%)	1,857 (30.2%)
Coping on present income	1,745 (47.2%)	608 (47.7%)	567 (48.2%)	2,920 (47.5%)
Finding it difficult	569 (15.4%)	262 (20.6%)	217 (18.4%)	1,048 (17.0%)
Finding it very difficult	161 (4.4%)	69 (5.4%)	71 (6%)	301 (4.9%)
Prefer not to say	15 (0.4%)	3 (0.2%)	4 (0.3%)	22 (0.4%)
Marital status				
Single	969 (26.2%)	334 (26.2%)	350 (29.7%)	1,653 (26.9%)
Married/living together	2,504 (67.7%)	849 (66.6%)	757 (64.3%)	4,110 (66.9%)
Widowed/partner has died	29 (0.8%)	11 (0.9%)	8 (0.7%)	48 (0.8%)
Divorced/separated	195 (5.3%)	80 (6.3%)	62 (5.3%)	337 (5.5%)
Work sector				
Health/social/beauty care	1,879 (50.8%)	372 (29.2%)	242 (20.6%)	2,493 (40.5%)
Other	1,818 (49.2%)	902 (70.8%)	935 (79.4%)	3,655 (59.5%)
Health conditions				
Migraine	690 (18.7%)	268 (21%)	200 (17%)	1,158 (18.8%)
Hypertension	663 (17.9%)	245 (19.2%)	149 (12.7%)	1,057 (17.2%)
High cholesterol/triglyceride	707 (19.1%)	223 (17.5%)	167 (14.2%)	1,097 (17.8%)
Acute episodes of atopic diseases	403 (10.9%)	157 (12.3%)	119 (10.1%)	679 (11%)
Type II diabetes	160 (4.3%)	80 (6.3%)	48 (4.1%)	288 (4.7%)
Gallstones	140 (3.8%)	60 (4.7%)	28 (2.4%)	228 (3.7%)
Angina pectoris	124 (3.4%)	68 (5.3%)	35 (3%)	227 (3.7%)
Kidney stones	121 (3.3%)	61 (4.8%)	33 (2.8%)	215 (3.5%)
Immunodeficiency syndrome	109 (2.9%)	57 (4.5%)	34 (2.9%)	200 (3.3%)
Gout	106 (2.9%)	55 (4.3%)	24 (2%)	185 (3%)
COPD	100 (2.7%)	61 (4.8%)	32 (2.7%)	193 (3.1%)
Myocardial infarction	79 (2.1%)	42 (3.3%)	21 (1.8%)	142 (2.3%)
Cirrhosis	51 (1.4%)	30 (2.4%)	13 (1.1%)	94 (1.5%)
Country				
Austria	734 (19.9%)	127 (10.0%)	115 (9.8%)	976 (15.9%)
Denmark	410 (11.1%)	132 (10.4%)	136 (11.6%)	678 (11.0%)
Germany	507 (13.7%)	161 (12.6%)	116 (9.9%)	784 (12.8%)
Greece	393 (10.6%)	185 (14.5%)	154 (13.1%)	732 (11.9%)
Italy	401 (10.8%)	79 (6.2%)	128 (10.9%)	608 (9.9%)
Poland	398 (10.8%)	208 (16.3%)	199 (16.9%)	805 (13.1%)
Spain	403 (10.9%)	165 (13.0%)	160 (13.6%)	728 (11.8%)
The Netherlands	421 (11.4%)	207 (16.2%)	159 (13.5%)	787 (12.8%)
Other	30 (0.8%)	10 (0.8%)	10 (0.8%)	50 (0.8%)

Data are presented as mean (SD) for continuous variables and *n* (%) for categorical variables.

Participants worked an average of 39.2 (6.1) hours per week and had been in their current job for 10.7 (SD: 9.6) years. Sixty percent were current night shift workers, 21% former, and 19% day workers. Current night shift workers reported a mean of 10.9 (9.5) years of night shift exposure, with the majority (59%) having more than 6 years of experience, and 70% were working rotating night shift schedules. Former night shift workers averaged 5.6 (6.3) years of night shift exposure, with 47% having worked night shifts for 1–5 years, and 58% had worked night shifts within the past 5 years.

### OW/OB by Night Shift Work Exposure

Overall, 32% of participants were identified with overweight and 17% with obesity, based on self-reported BMI solely, indicating that approximately half of the participants had a BMI ≥25 kg/m^2^. Regardless of shift work, Germany (61%) had the highest prevalence of OW/OB, while Denmark (41%) had the lowest (Fig. 1). Night shift exposure was associated with elevated odds of OW/OB: former night shift workers showed the highest prevalence of OW/OB (53%), with significantly greater odds (OW: OR 1.55; 95% CI: 1.28–1.87; OB: OR: 1.51; 95% CI: 1.19–1.93) compared to day workers (42%), while current night shift workers (51%) also had elevated odds (OW: OR 1.33; 95% CI: 1.15–1.58; OB: OR 1.62; 95% CI: 1.33–1.99), after adjusting for potential confounders (Fig. 2, online suppl. Fig. 2a, b). Across countries, the highest OW/OB prevalence was observed among current night shift workers in Poland (69%), and among former night shift workers (66%) and day workers (53%) in Germany (Fig. 1). Gender patterns were consistent among current and former night shift workers, with men showing a higher prevalence than women (current night shift workers: 59% vs. 44%; former night shift workers: 58% vs. 48%; day workers: 49% vs. 37%).

**Fig. 1.**
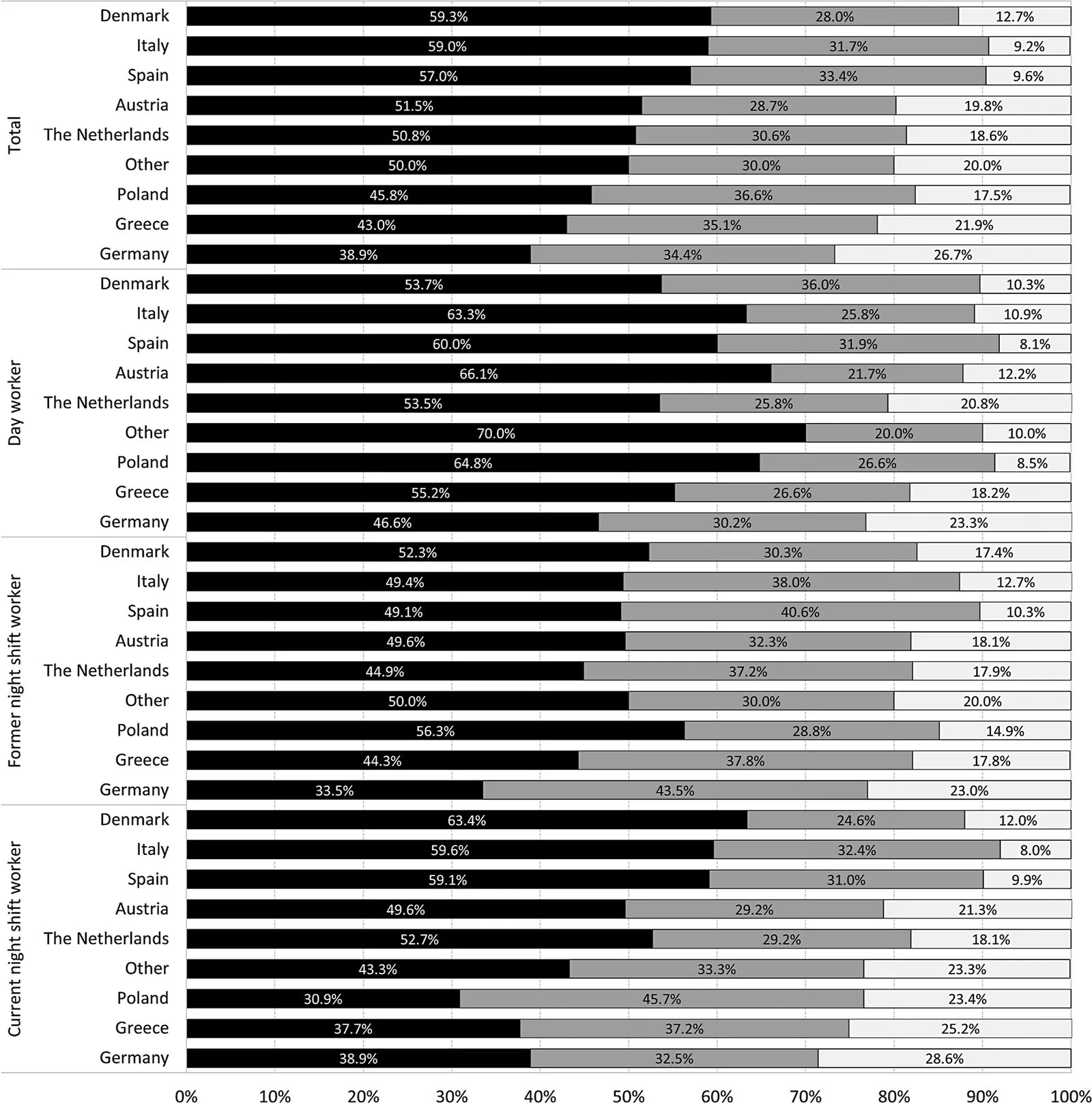
Prevalence of BMI categories by night shift work exposure (total participants, day workers, former night shift workers, current night shift workers) in European countries (Denmark, Italy, Spain, Austria, The Netherlands, Other, Poland, Greece, Germany). Black: normal weight (BMI <24.9 kg/m^2^); dark grey: overweight (BMI 25.0–29.9 kg/m^2^); light grey: obesity (BMI ≥30 kg/m^2^).

**Fig. 2.**
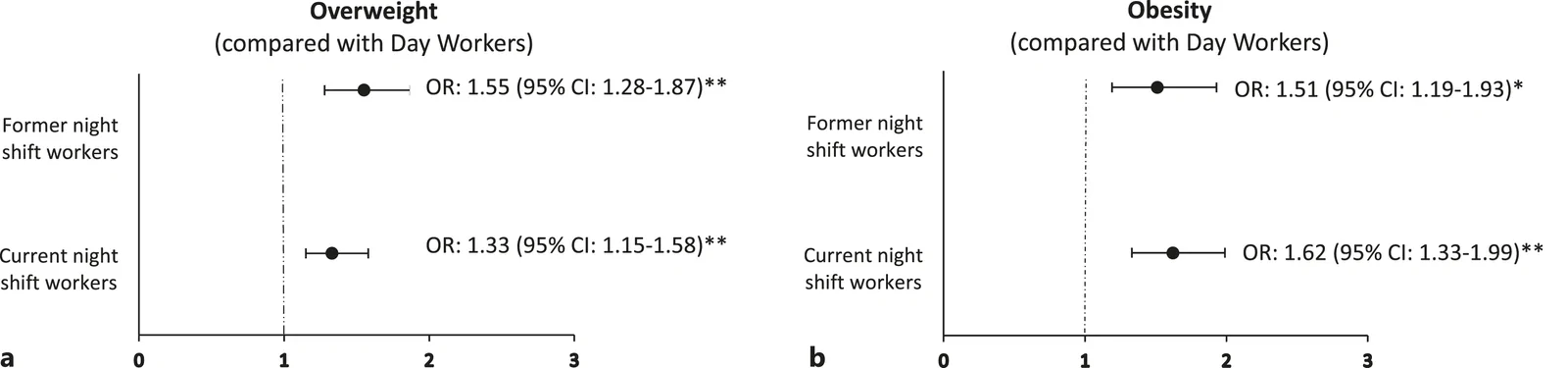
Forest plots showing adjusted ORs and 95% CIs for overweight (**a**) and obesity (**b**) by night shift work exposure (former and current night shift workers) compared to day workers. Results are based on logistic regression adjusted for age, gender, countries, education level, income satisfaction, marital status, weekly working hours, and duration of current job. OR, odds ratio; CI, confidence interval.

Logistic regression analyses of working schedule characteristics showed that among current night shift workers, longer cumulative exposure was associated with progressively higher odds of both OW/OB compared with day workers (Table 2). Those with more than 12 years of night shift exposure had 62% higher odds of overweight (OR 1.62; 95% CI: 1.31–2) and more than twice the odds of obesity (OR 2.31; 95% CI: 1.77–3). A similar pattern was observed for monthly frequency of night shifts, with workers performing more than 7 shifts per month showing elevated odds of overweight (OR 1.35; 95% CI: 1.11–1.63) and higher odds of obesity (OR 1.77; 95% CI: 1.4–2.25). Rotating night shifts were also associated with higher odds of overweight (OR 1.44; 95% CI: 1.22–1.7) and obesity (OR 1.71; 95% CI: 1.38–2.12). Among former night shift workers, comparable patterns were observed. Furthermore, a longer time since the last night shift remained associated with elevated odds of obesity (OR 4.16; 95% CI: 2.28–7.62).

**Table 2.** Association between night shift exposure and metrics with OW/OB

​	Current night shift workers vs. day workers				Former night shift workers vs. day workers			
*Ref.: normal weight*	*%*	overweight *OR (95% CI)*	*%*	obesity *OR (95% CI)*	*%*	overweight *OR (95% CI)*	*%*	obesity *OR (95% CI)*
Years of night shift exposure (tertile)								
Day workers	28	Ref.	13.7	Ref.	28.0	Ref.	13.7	Ref.
0–4.9 years	29.5	**1.25 (1.02–1.52)**	13.5	1.19 (0.92–1.55)	33.5	**1.4 (1.14–1.72)**	14.4	1.23 (0.94–1.62)
5–11.9 years	29.8	1.17 (0.96–1.42)	17.7	**1.46 (1.14–1.86)**	43.4	**1.97 (1.45–2.68)**	18	**1.74 (1.17–2.58)**
>12 years	37.4	**1.62 (1.31–2)**	25.0	**2.31 (1.77–3)**	38.9	1.73 (0.98–3.08)	36.8	**4.16 (2.28–7.62)**
Frequency of night shifts/months (tertile)								
Day workers	28.0	Ref.	13.7	Ref.	​	​	​	​
0–2.9 night shifts/months	31.1	1.19 (0.97–1.45)	18.0	**1.44 (1.12–1.85)**	​	​	​	​
3–6.9 night shifts/months	32.1	**1.48 (1.22–1.8)**	17.9	**1.62 (1.26–2.07)**	​	​	​	​
>7 night shifts/months	33.8	**1.35 (1.11–1.63)**	21.0	**1.77 (1.4–2.25)**	​	​	​	​
Direction of shift rotation								
Day workers	28.0	Ref.	13.7	Ref.	28.0	Ref.	13.7	Ref.
Fixed night shift	30.9	1.12 (0.92–1.36)	17.0	**1.32 (1.02–1.7)**	34.2	**1.33 (1.08–1.64)**	14.4	1.21 (0.92–1.6)
Rotating night shift	32.9	**1.44 (1.22–1.7)**	19.6	**1.71 (1.38–2.12)**	39.5	**2.03 (1.56–2.65)**	21.4	**2.07 (1.49–2.87)**
Years since last night shift (tertile)								
Day workers	​	​	​	​	28.0	Ref.	13.7	Ref.
0–1.9 years	​	​	​	​	33.4	**1.64 (1.26–2.15)**	16.8	1.7 (1.21–2.38)
2–5.9 years	​	​	​	​	33.3	**1.4 (1.08–1.83)**	15.8	1.35 (0.96–1.91)
>6 years	​	​	​	​	41.2	**1.56 (1.20–2.02)**	17.1	1.32 (0.94–1.86)

Results are based on multivariate logistic regression adjusted for age, gender, countries, education level, income satisfaction, marital status, weekly working hours, and duration of current job and are presented as ORs with 95% CIs. Statistically significant results are shown in bold.

### Lifestyle and Psychosocial Parameters and OW/OB by Night Shift Work Exposure

Descriptive information on lifestyle and psychosocial parameters of current and former night shift workers as well as day workers is shown in online supplementary Table 1. Results of the logistic regression analyses show that among current and former night shift workers, fast eating rate, physical inactivity, and poor mental well-being (only among current night shift workers) were significantly associated with higher odds of OW/OB, with a small-to-medium range of effect sizes. Among current night shift workers, less favourable dietary pattern was additionally associated with higher odds of obesity (OR 1.66; 95% CI: 1.37–2.01; Table 3). Further analyses on dietary patterns indicated that current night shift workers with a high intake of sugar-sweetened beverages (OR 1.75, 95% CI: 1.38–2.2), low intake of whole grains (OR 1.41, 95% CI: 1.17–1.71), legumes (OR 1.57, 95% CI: 1.26–1.95), vegetables (OR 1.39, 95% CI: 1.14–1.7), and fruits (OR 1.66, 95% CI: 1.36–2.03) showed higher odds of obesity compared to those with more favourable dietary patterns. Notably, among current night shift workers, moderate-to-high alcohol consumption was inversely associated with OW/OB (OW: OR 0.83; 95% CI: 0.7–0.97; OB: OR 0.81; 95% CI: 0.66–0.99), and a similar inverse association with obesity was observed among former night shift workers (OR 0.62, 95% CI: 0.42–0.89). In contrast, among day workers, fewer lifestyle factors were significantly associated with weight status: fast eating rate and physical inactivity were associated with overweight while poor mental well-being was related to obesity (Table 3).

**Table 3.** Association between lifestyle parameters and OW/OB, stratified by night shift work exposure

​	Current night shift workers		Former night shift workers		Day workers	
*Ref.: normal weight*	overweight *n* = 1,196 *OR (95% CI)*	obesity *n* = 697 *OR (95% CI)*	overweight *n* = 458 *OR (95% CI)*	obesity *n* = 213 *OR (95% CI)*	overweight *n* = 330 *OR (95% CI)*	obesity *n* = 161 *OR (95% CI)*
Fast eating rate *(ref. slow/average)*	**1.28 (1.09, 1.50)**	**1.66 (1.37, 2.01)**	**1.37 (1.04, 1.8)**	**1.55 (1.09, 2.2)**	**1.64 (1.22, 2.2)**	1.26 (0.86, 1.85)
Less favourable dietary pattern *(ref. more favourable)*	1.05 (0.9, 1.24)	**1.71 (1.39, 2.1)**	0.83 (0.63, 1.09)	0.86 (0.6, 1.23)	**1.5 (1.12, 2.02)**	1.01 (0.7, 1.46)
Physical inactivity *(ref. active)*	**1.45 (1.2, 1.77)**	**2.23 (1.8, 2.78)**	**1.48 (1.07, 2.05)**	**1.93 (1.31, 2.86)**	1.29 (0.91, 1.84)	1.52 (0.99, 2.32)
Severe insomnia problems *(ref. no/mild/moderate)*	0.88 (0.75, 1.03)	1.1 (0.91, 1.34)	1.02 (0.79, 1.32)	1.03 (0.73, 1.44)	1.06 (0.8, 1.4)	1.39 (0.97, 2)
Moderate/high alcohol consumption *(ref. no/low)*	**0.83 (0.7, 0.97)**	**0.81 (0.66, 0.99)**	1 (0.77, 1.32)	**0.62 (0.42, 0.89)**	1.13 (0.84, 1.52)	0.78 (0.52, 1.17)
Current smokers *(ref. never)*	**0.8 (0.67, 0.96)**	1.06 (0.85, 1.34)	1.06 (0.78, 1.45)	0.74 (0.49, 1.1)	0.84 (0.6, 1.17)	0.85 (0.55, 1.31)
High perceived stress level *(ref. no/low)*	0.89 (0.75, 1.05)	0.99 (0.8, 1.23)	1.07 (0.8, 1.44)	1.05 (0.71, 1.53)	0.97 (0.71, 1.32)	0.89 (0.59, 1.33)
Poor mental well-being *(ref. good)*	**1.18 (1, 1.39)**	**1.27 (1.04, 1.54)**	1.24 (0.94, 1.63)	1.13 (0.79, 1.61)	1.08 (0.79, 1.46)	**1.51 (1.04, 2.19)**

Results are presented as ORs with 95% CIs. Statistically significant results are shown in bold.

Logistic regression adjusted for age, gender, countries, education level, income satisfaction, marital status, weekly working hours, and duration of current job.

Adjusting the association between night shift exposure and OW/OB additionally for lifestyle factors and psychosocial factors resulted in only minor changes to the observed associations. When current smoking was additionally included in the model, the ORs for OW/OB increased slightly among both current and former night shift workers (Table 4).

**Table 4.** Association between night shift work exposure and OW/OB, adjusted for lifestyle factors

​	Current night shift workers		Former night shift workers	
*Ref.: day workers*	overweight *n* = 1,196 *OR (95% CI)*	obesity *n* = 697 *OR (95% CI)*	overweight *n* = 458 *OR (95% CI)*	obesity *n* = 213 *OR (95% CI)*
Model 0 (unadjusted)	**1.39 (1.19, 1.62)**	**1.64 (1.35, 1.99)**	**1.59 (1.33, 1.91)**	**1.49 (1.18, 1.89)**
Model 1 (adjusted)^a^	**1.33 (1.13, 1.56)**	**1.58 (1.29, 1.94)**	**1.53 (1.26, 1.85)**	**1.55 (1.14, 1.86)**
Additional adjustments^b^				
Fast eating rate *(vs. slow/average)*	**1.31 (1.12, 1.54)**	**1.53 (1.25, 1.89)**	**1.52 (1.26, 1.84)**	**1.45 (1.13, 1.85)**
Less favourable dietary pattern *(vs. more favourable)*	**1.33 (1.13, 1.55)**	**1.54 (1.25, 1.89)**	**1.53 (1.26, 1.84)**	**1.46 (1.15, 1.87)**
Physical inactivity *(vs. active)*	**1.33 (1.14, 1.56)**	**1.59 (1.29, 1.95)**	**1.53 (1.27, 1.85)**	**1.46 (1.14, 1.87)**
Severe insomnia problems *(vs. no/mild/moderate)*	**1.35 (1.15, 1.58)**	**1.56 (1.27, 1.92)**	**1.52 (1.26, 1.84)**	**1.45 (1.13, 1.85)**
Moderate/higher alcohol consumption *(vs. no/low)*	**1.33 (1.14, 1.56)**	**1.59 (1.3, 1.96)**	**1.52 (1.26, 1.84)**	**1.47 (1.15, 1.88)**
Current smokers (*vs. never)*	**1.41 (1.18, 1.69)**	**1.62 (1.28, 2.06)**	**1.57 (1.26, 1.95)**	**1.60 (1.21, 2.13)**
High perceived stress level (*vs. no/low*)	**1.33 (1.13, 1.56)**	**1.58 (1.29, 1.95)**	**1.53 (1.26, 1.85)**	**1.46 (1.14, 1.86)**
Poor mental well-being *(vs. good)*	**1.32 (1.12, 1.54)**	**1.57 (1.28, 1.93)**	**1.52 (1.26, 1.84)**	**1.46 (1.15, 1.87)**

Results are presented as ORs with 95% CIs. Statistically significant results are shown in bold.

^a^ Logistic regression adjusted for age, gender, countries, education level, income satisfaction, marital status, weekly working hours, and duration of current job.

^b^ Lifestyle/psychosocial factors were additionally entered into the model one at a time; the OR for OB/OB is presented.

### Sensitivity Analyses

There was a strong agreement between the single question and the detailed schedule-based classification (Cohen’s κ = 0.9, *p* < 0.001; 6.2% discrepant). Sensitivity analyses using the schedule-based classification confirmed consistent associations with OW/OB, supporting the robustness of the findings (*results not shown*).

## Discussion

Across European countries, approximately half of night shift workers (current and former) were living with OW/OB (identified by BMI <25 kg/m^2^), indicating a higher prevalence compared with day workers. Regardless of night shift work, the highest prevalence of OW/OB was observed in Germany. Regarding working schedule metrics, longer night shift exposure, higher monthly frequency, and rotating schedules were consistently associated with higher odds of OW/OB compared with day work. Fast eating rate, less favourable dietary patterns, physical inactivity, and poor mental well-being were associated with a higher likelihood of OW/OB in current night shift workers.

Overall, the prevalence of OW/OB was particularly noticeable given the high educational level in our sample, suggesting that rates could be even higher in populations with lower socio-economic status [37]. Apart from that, significant variations were observed across Europe. Compared with the prevalence data from the Global Burden of Disease Study (2021), slightly higher values were observed in Germany, whereas lower ones were found in Austria, the Netherlands, Greece, Italy, Denmark, Poland, and Spain (with differences ranging from 6 to 20%) [1]. These discrepancies are likely influenced by the characteristics of our sample, which was not representative of the country’s population and in which 44% held a university degree, oversampling of night shift workers. The observed differences between countries may also reflect variations in local contextual factors, including social class composition, working environments, and other country-specific influences (e.g., national dietary patterns, lifestyle behaviours, and public health policies). However, these mechanisms were not directly measured in the present study, and individual perceptions of, and reporting on, the magnitude of effects may differ. Therefore, the country-specific findings should be interpreted as contextual differences rather than as fully explained effects. Regarding gender, the prevalence of OW/OB was generally higher among men than women, a pattern that is also observed in the Global Burden of Disease study [1].

We observed a 62% increase in the odds of obesity with current night shift work, compared to day work. This is in line with previous literature but a stronger association compared to previous studies. For example, Sun et al. [3] reported a 23% increase in the odds of OW/OB among night shift workers, and an Italian study found only a 6% increase in obesity [38]. Our estimates were also higher than those reported by Liu et al. [5], who observed a 17% increased relative risk of obesity and a 25% increased relative risk of overweight, although direct comparisons are limited by differences in effect measures (ORs vs. relative risks).

Notably, our data also show a high prevalence of OW/OB among former night shift workers. This observation is particularly interesting as 35% of this group had not worked night shifts for ≥6 years. Our estimates for former workers are comparable to those reported in the meta-analysis by Sun et al. [3], which found a 24% increased risk of obesity in men and a 34% increased risk in women. However, results are in line with findings from a recent longitudinal study, which reported that BMI remained higher after cessation of night shift work compared with controls, indicating lasting effects of prior exposure [39]. More longitudinal studies following night shift workers after cessation of night work and account for time since cessation of shift work would be informative to investigate the mechanisms underlying these long-lasting effects of circadian disruption.

Among working schedule characteristics, longer night shift exposure, higher monthly frequency, and rotating schedules were consistently associated with higher odds of OW/OB compared with day work, indicating a dose-response relationship. These findings are in line with the existing literature, as meta-analyses have reported an 18–21% higher relative risk of OW/OB among rotating night shift workers [40]. Moreover, previous studies have described gradual increases in BMI and a higher risk of OW/OB with longer duration of night shift work and greater shift frequency [7, 41–43].

Importantly, we found associations not only with work schedule characteristics but also with various lifestyle and psychosocial factors. Fast eating rate was consistently associated with higher odds of OW/OB across all groups, aligning with previous research [44]. Further, less favourable dietary patterns, including high sugar-sweetened beverage intake and low consumption of whole grains, legumes, vegetables, and fruits, were associated with higher odds of obesity among night shift workers, consistent with European data [45, 46]. Although it is widely recognized that fruit intake is part of a healthy diet, the relationship between fruit consumption and OW/OB is complex. Higher intakes of fruit juice have been associated with increased visceral adiposity [47]. However, evidence suggests that eating whole fruit is unlikely to contribute to excess energy intake and adiposity [48, 49]. In our study, participants were only asked about their consumption of whole fruit (fresh or cooked), excluding fruit juices. Physical inactivity also showed a significant inverse association with OW/OB, suggesting that lower physical activity is a general risk factor, as supported by large epidemiological studies [50]. Also, more than three glasses of alcohol per week was associated with lower odds of OW/OB among current and former night shift workers, but not in day workers; this aligns with previous literature, suggesting a potential link between alcohol intake and OW/OB risk, although findings are mixed [51]. Alcohol consumption was assessed using an ordinal item and subsequently dichotomized, which may have reduced the granularity of information and limited the ability to capture subtle differences in drinking patterns. Finally, the association of poor mental health/well-being with increased prevalence OW/OB was only significant in current night shift workers and not in former night shift and day workers. This may be due to lower statistical power in former night shift and day worker subgroups.

When associations between night shift exposure and OW/OB were sequentially adjusted for lifestyle/psychosocial factors, only minor changes were observed, suggesting that these factors explain a small part of the observed relationships. Adjusting for smoking slightly increased the ORs, indicating possible negative confounding which may partially mask the association between night shift work and body weight. These findings contrast with a Danish cross-sectional study, which found that physical inactivity, poor diet, and poor sleep partly explained the link between shift work and obesity, while smoking was associated with lower odds of obesity [52]. From a public health perspective, our findings imply that interventions focusing solely on lifestyle behaviours may be insufficient to reduce the risk of OW/OB among current and also former night shift workers. Structural and occupational approaches addressing working time arrangements are needed. In addition to such interventions, a qualitative study of UK healthcare night shift workers highlighted environmental and social influences, such as limited access to healthy food, inadequate storage, and time constraints, on dietary patterns, suggesting that effective interventions should include food environment redesign [53].

The study has several strengths. Firstly, it included detailed information on night shift work metrics and evaluated both current night shift workers as well as former night shift workers, and day workers. Secondly, the broad geographic representation across multiple European countries provides a unique opportunity to examine associations between night shift exposure, lifestyle factors, and OW/OB across Europe. Thirdly, the inclusion of night shift workers from diverse sectors enhances the generalizability of the findings. However, the study also has some limitations. Firstly, data were collected via an online survey, which may have introduced information bias, as the outcomes were self-reported, and this may have led to an underestimation of BMI [54]. Secondly, the response rate was low, and as the sample predominantly consisted of highly educated individuals, selection bias is assumed to be present. Thirdly, the cross-sectional design limits the ability to make inferences about the direction of the association. Fourthly, based on Cohen’s guidelines for interpreting ORs [55, 56], most of the observed associations were in the small-to-medium range (OR <1.55: small; OR 1.55 −<2.5: medium). Only the association between former night shift workers, compared with day workers, and obesity after more than 12 years of night shift exposure showed a large-sized association (OR 2.5 −<4). However, even small associations may be relevant from a public health perspective, particularly for a common exposure such as night shift work [40, 57]. Fifthly, the new framework for the diagnosis, staging, and management of obesity in adults, published by the European Association for the Study of Obesity (EASO), which considers waist-to-height ratio and medical, functional, or psychological impairments [58], could not be applied due to the self-reported methodology. To address these aforementioned limitations, a multicentric cross-sectional study has been designed to better elucidate obesogenic risk factors, eating habits, and sleep patterns among day and night shift workers in Europe [59]. This study will include objective anthropometric measurements, assessments of sensory perception and food preference assessments, and comprehensive questionnaires. Biological samples (including blood, hair, urine, and faeces) will also be collected.

## Conclusion

Current and former night shift workers have higher odds of OW/OB, identified by self-reported BMI, compared with day workers across European countries and occupations. These increased odds are associated with a broad range of work-related factors, including rotating shift schedules, a greater number of night shifts per month, and longer exposure to night shifts, as well as lifestyle and psychosocial factors such as a fast eating rate, physical inactivity, unfavourable dietary patterns, and poor mental well-being. While these lifestyle behaviours and psychosocial factors only partially account for these increased odds, our findings highlight the importance of considering broader structural, environmental, and occupational factors in future research and prevention initiatives. This is important given that elevated body weight may persist even years after night shift work has ceased.

## Key Points

- Both current and former night shift workers had higher prevalence of OW/OB than day workers.
- Rotating schedules, a greater number of night shifts per month, and longer exposure to night shifts as well as a fast eating rate, physical inactivity, unfavourable dietary patterns, and poor mental well-being were associated with OW/OB.
- Sequential adjustment of the association between shift work exposure and OW/OB for lifestyle factors showed that those factors explain only a small part of the increased risk of OW/OB, highlighting the need for targeted prevention strategies and policy action for night shift workers.

## Acknowledgments

We would like to thank EIT Health and the company synoint for their valuable support in participant recruitment, and Kilian Gandolf for designing the survey in REDCap.

## Statement of Ethics

This study was performed in accordance with the Declaration of Helsinki [60]. This human study was approved by Wageningen University & Research (2024-001) – covering all countries, the Medical University of Vienna (1152/2024) – Austria; and Universität Bremen 89 (2024-04) – Germany; no approval was needed for Poland according to the Bioethics Committee of Medical University of Lodz (opinion no. RNN/63/24/KN, February 13, 2024) – Poland and Denmark, according to the local regulations. Ethical approval granted by Wageningen University & Research covered Greece, Italy, and Spain, so no additional national ethical approvals were required in these countries. Participation in the study was voluntary. Written informed consent was provided by all participants electronically before completing the online survey, and they could stop the survey at any time.

## Conflict of Interest Statement

The authors have no conflicts of interest to declare.

## Funding Sources

This research was funded by the European Union under the Horizon Europe call “HORIZON-HLTH-2022-STAYHLTH-01-05.” The funder had no role in the design, data collection, data analysis, and reporting of this study.

## Author Contributions

K.H.W., M.H.V., K.P., M.U., S.H., K.H., M.A.W., M.W., H.J., H.L., I.M., B.F.N., and E.F. designed the study. M.H.V., S.H., K.B.O., J.R., M.A.W., C.D., H.L., I.M., M.U., A.L.A., C.H., B.F.N., D.G.M., C.C., K.H.W., V.S., K.S., and U.K. coordinated or did the data collection. S.H., E.W., M.W., K.P., L.S., H.B., and I.S. planned and performed the statistical analyses and drafted the manuscript. All authors have interpreted, reviewed, and approved the manuscript for publication.

## Data Availability

All data generated or analysed during this study are included in this article. Further enquiries can be directed to the corresponding author.
